# Paclobutrazol Ameliorates Low-Light-Induced Damage by Improving Photosynthesis, Antioxidant Defense System, and Regulating Hormone Levels in Tall Fescue

**DOI:** 10.3390/ijms23179966

**Published:** 2022-09-01

**Authors:** Bowen Liu, Si Long, Kening Liu, Tianqi Zhu, Jiongjiong Gong, Shuanghong Gao, Ruijia Wang, Liyun Zhang, Tieyuan Liu, Yuefei Xu

**Affiliations:** College of Grassland Agriculture, Northwest A&F University, Yangling, Xianyang 712100, China

**Keywords:** antioxidant system, hormone levels, low-light tolerance, paclobutrazol, photosynthesis, tall fescue

## Abstract

Paclobutrazol (PBZ) is a plant-growth regulator (PGR) in the triazole family that enhances plant tolerance to environmental stresses. Low-light (LL) intensity is a critical factor adversely affecting the growth of tall fescue (*Festuca arundinacea* Schreb.). Therefore, in this study, tall fescue seedlings were treated with PBZ under control and LL conditions to investigate the effects of PBZ on enhancing LL stress resistance by regulating the growth, photosynthesis, oxidative defense, and hormone levels. Our results reveal that LL stress reduced the total biomass, chlorophyll (Chl) content, photosynthetic capacity, and photochemical efficiency of photosystem II (PSII) but increased the membrane lipid peroxidation level and reactive oxygen species (ROS) generation. However, the application of PBZ increased the photosynthetic pigment contents, net photosynthetic rate (*P*_n_), maximum quantum yield of PSII photochemistry (*F*_v_/*F*_m_), ribulose-1,5-bisphosphate carboxylase (RuBisCO) activity, and starch content. In addition, PBZ treatment activated the antioxidant enzyme activities, antioxidants contents, ascorbate acid-glutathione (AsA-GSH) cycle, and related gene expression, lessening the ROS burst (H_2_O_2_ and O_2_∙^−^). However, the gibberellic acid (GA) anabolism was remarkably decreased by PBZ treatment under LL stress, downregulating the transcript levels of kaurene oxidase (KO), kaurenoic acid oxidase (KAO), and GA 20-oxidases (GA20ox). At the same time, PBZ treatment up-regulated 9-*cis*-epoxycarotenoid dioxygenase (NCED) gene expression, significantly increasing the endogenous abscisic acid (ABA) concentration under LL stress. Thus, our study revealed that PBZ improves the antioxidation and photosynthetic capacity, meanwhile increasing the ABA concentration and decreasing GA concentration, which ultimately enhances the LL stress tolerance in tall fescue.

## 1. Introduction

Light energy from solar radiation is essential for photosynthesis, growth, and development in plants [1]. Turfgrass is the leading ornamental plant with great aesthetic value and contributes to landscape beauty. However, limited light availability causes low-light (LL) stress in turfgrass, adversely affecting its growth and resulting in turfgrass decline [2,3]. Low-light intensity influences plant morphology, photosynthetic physiology, and biochemical and phytohormone levels [4,5,6]. In turfgrass, the turf quality, color, and density of tall fescue decrease under 92% LL stress caused by tree shading [7]. In addition, the dry weight and photosynthetic traits of cool-season turfgrass species decrease with the increase in shade intensity [8]. Plants in low-light areas have a slower growth rate and produce fewer tillers to utilize light more efficiently. Furthermore, plants could survive under LL stress by activating antioxidant enzymes and accumulating non-enzymatic antioxidants to alleviate reactive oxygen species (ROS) damage [9]. Enzymatic antioxidants include superoxide dismutase (SOD), peroxidase (POD), catalase (CAT), ascorbate peroxidase (APX), and glutathione reductase (GR), while non-enzymatic antioxidants comprise total phenolics, flavonoids, carotenoids (Car), glutathione (GSH), and ascorbate acid (AsA) [10].

Plant-growth regulators (PGRs) are natural substances or synthetic chemical compounds that regulate the plant’s physiological response when applied in small amounts [11]. Paclobutrazol (PBZ), a triazole family with PGR properties, inhibits shoot elongation inhibition while stimulating root growth by inhibiting gibberellic acid (GA) synthesis [12]. Therefore, it has the potential to maintain turfgrass at an appropriate height, which reduces the cost of mowing, especially during the plant’s rapid growth period [13]. Additionally, PBZ alleviates abiotic stress by regulating several physiological and biochemical processes [14]. Furthermore, PBZ crosstalk with abscisic acid (ABA) signals alleviates oxidative burst in rice (*Oryza sativa* L.) under drought stress [15]. The application of PBZ also significantly elevates the total antioxidant activity, total phenolic content, and protein oxidation, scavenging the excessive ROS, while increased ABA content activates the antioxidant system. In mung beans [*Vigna radiata* (L.) Wilczek], PBZ alleviates the decreased leaf chlorophyll and membrane stability induced by stress conditions [16]. However, the impacts of PBZ on photosynthetic capacity and the ascorbate-acid–glutathione (AsA-GSH) cycle under environmental stress are less studied. With respect to LL stress, previous studies mainly reported the effects of PBZ on developmental processes and primary morphological changes, meanwhile, only trees and horticultural plants are used as research objects. Therefore, the role of PBZ in comprehensively regulating morphological, physiological, and gene-expression levels in tall fescue under LL stress is still unclear.

Tall fescue is a cool-season turfgrass cultivated in northern China for aesthetic value and microclimate improvement. Unfortunately, tree canopies and buildings create a detrimental low-lighting microenvironment, restricting tall-fescue growth [17]. The density of the tree shade depends on the time of year. For example, deciduous trees could produce severe LL stress in summer; moreover, turfgrass in cities is subjected to permanent low-light because of high buildings, both of which exacerbate the competition for light and make tall fescue weak and sparse. Therefore, the restriction of GA biosynthesis by PGRs (including PBZ) improves turf quality while enhancing LL tolerance under the LL condition [18]. Although studies have proven the application of PBZ on plants promotes abiotic stress tolerance [14,15], the involvement of PBZ in LL stress, especially the specific mechanisms at photosynthesis, the antioxidant defense system, plant endogenous hormones, and related gene-expression levels, by which PBZ regulates LL tolerance, remain unclear. Therefore, this study aims to elucidate the important role of PBZ in alleviating LL stress and its regulatory mechanisms in tall fescue.

## 2. Results

### 2.1. Optimal PBZ Concentration and Morphological Characteristics

The application of different concentrations of PBZ alleviated the adverse effects of LL stress. Tall fescue treated with 200 mg L^−1^ PBZ recorded the highest plant height, leaf width, total biomass, total Chl content, and the least MDA content compared to the LL treatment (Table 1). Therefore, 200 mg L^−1^ was identified as the most suitable PBZ concentration for use in subsequent experiments.

**Figure 1:**
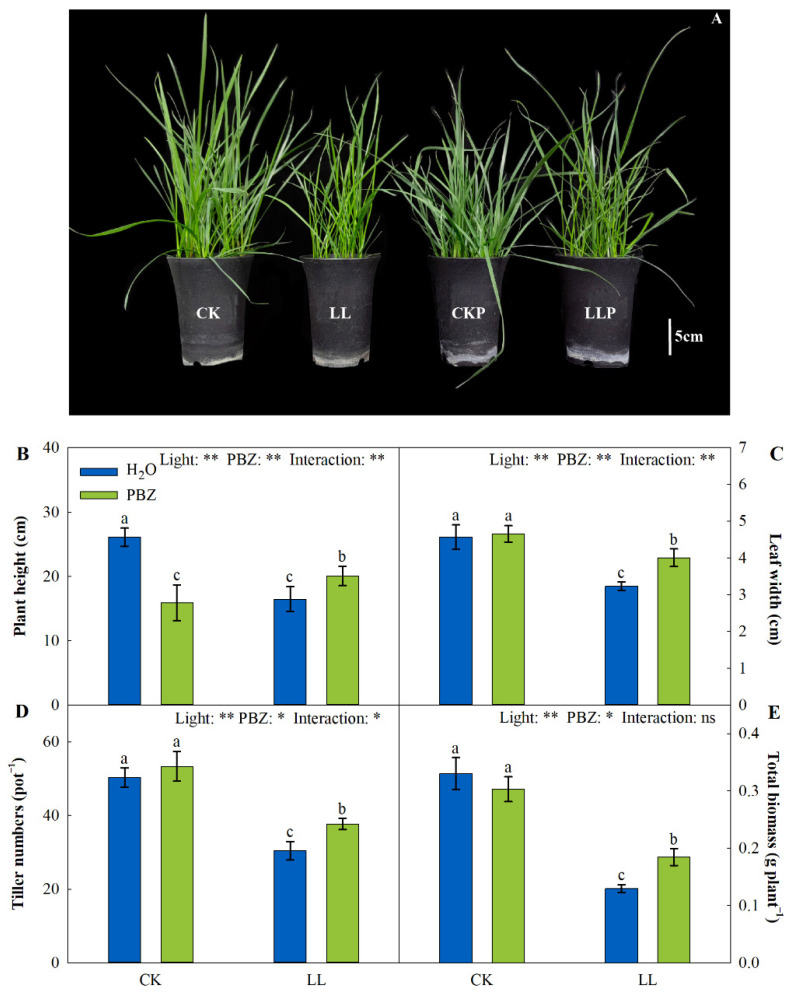
Effects of paclobutrazol on the morphology (**A**), plant height (**B**), leaf width (**C**), tiller numbers (**D**), and total biomass (**E**) of tall fescue under low-light (LL) stress. H_2_O and PBZ represent the treatments treated with distilled water and 200 mg L^−1^ paclobutrazol under control (CK) or LL stress, respectively. The data are mean ± SD values (n = 3). * and ** indicate significant differences at *p* < 0.05 and *p* < 0.01, respectively. ns indicates not significant. Different letters above the vertical bars indicate significant differences at *p* < 0.05 according to Duncan’s multiple range test.

The application of PBZ significantly decreased the plant height under the control condition. At the same time, in the LL treatment, the plant height, leaf width, tiller numbers, and total biomass were significantly decreased compared to the control plants. However, the simultaneous application of PBZ and LL stress markedly enhanced the morphological characteristics of the turfgrass (Figure 1).

### 2.2. Chlorophyll Content, Gas Exchange, and Chlorophyll Fluorescence Parameters

**Figure 2:**
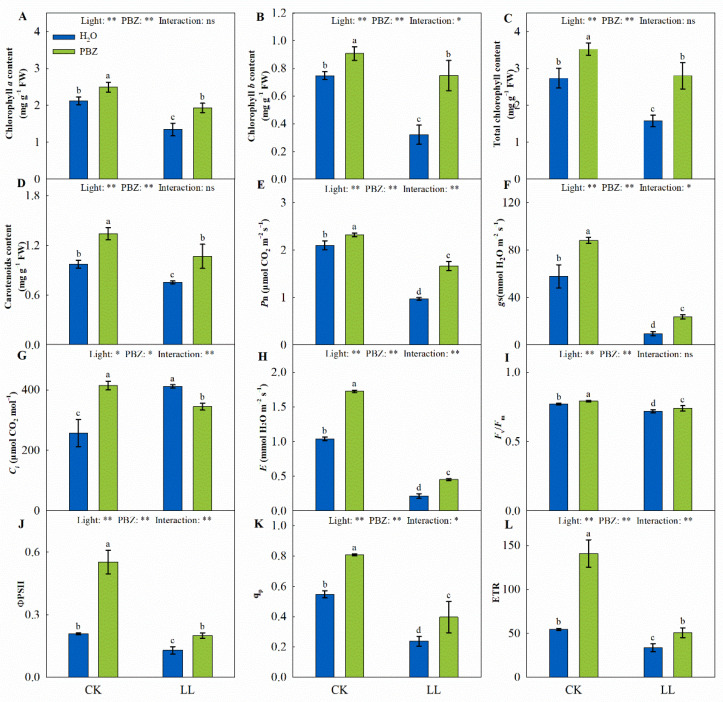
Levels of chlorophyll *a* content (**A**), chlorophyll *b* content (**B**), total chlorophyll content (**C**), carotenoids content (**D**), net photosynthetic rate (*P*_n_, (**E**)), stomatal conductance (*g*s, (**F**)), intercellular CO_2_ concentration (*C_i_*, **G**), transpiration rate (*E*, (**H**)), maximum quantum yield of photosystem II (PSII) photochemistry (*F*_v_/*F*_m_, (**I**)), actual quantum yield of PSII photochemistry (ΦPSII, (**J**)), photochemical quenching coefficient (q_p_, (**K**)), and relative electron transport rate (ETR, (**L**)) of tall fescue leaves induced by paclobutrazol under control (CK) and LL stress. * and ** indicate significant differences at *p* < 0.05 and *p* < 0.01, respectively. ns indicates not significant. Different letters above the vertical bars indicate significant differences at *p* < 0.05 according to Duncan’s multiple range test.

The chlorophyll concentration and photosynthesis parameters were significantly different under the influence of light and PBZ. Specifically, PBZ significantly increased the Chl *a*, Chl *b*, and total Chl concentrations by 1.17, 1.22, and 1.29 times and 1.65, 2.33, and 1.77 times, under control and LL conditions, respectively (Figure 2). The content of Car was decreased under LL stress, whereas PBZ application significantly increased the Car concentration (Figure 2D).

Except for *C_i_*, all gas-exchange parameters (*P*_n_, *g*s, and *E*) were reduced in LL-treated tall fescue seedlings. Specifically, LL treatment significantly decreased *P*_n_, *g*s, and *E* by 53.71%, 83.42%, and 79.41%, respectively, whereas *C_i_* was increased by 60.03% compared to the control (Figure 2). Additionally, the effect of PBZ on improving photosynthesis was more significant under LL stress.

The combined application of PBZ and LL stress significantly increased *F*_v_/*F*_m_, ΦPSII, q_p_, and ETR by 2.97%, 55.41%, 67.22%, and 50.35% relative to the application of LL stress and distilled water, respectively. In addition, *F*_v_/*F*_m_, ΦPSII, q_p_, and ETR were increased by 2.80%, 164.59%, 47.40%, and 158.28%, respectively, upon treatment with PBZ and the control (Figure 2I–L). These results revealed that PBZ treatment could mitigate the inhibition of photosynthesis caused by LL stress in tall fescue.

### 2.3. RuBisCO Activity and Photosynthates

**Figure 3:**
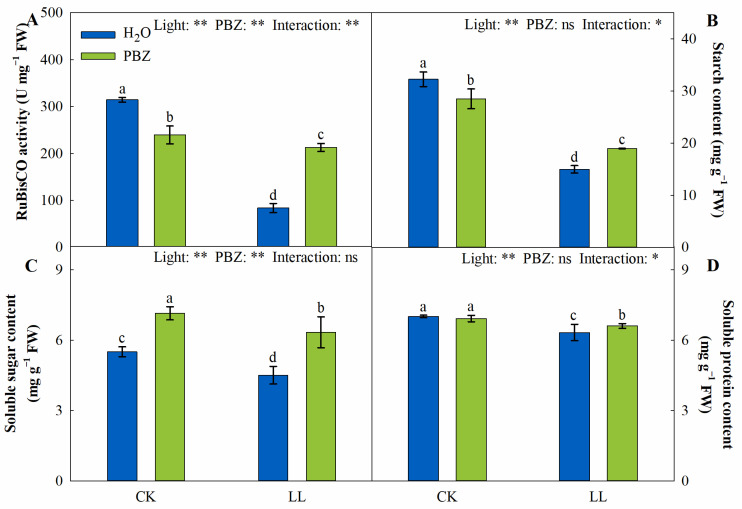
Changes in RuBisCO activity (**A**), starch content (**B**), soluble-sugar content (**C**), and soluble-protein content (**D**) of tall fescue leaves induced by paclobutrazol under control (CK) and LL stress. * and ** indicate significant differences at *p* < 0.05 and *p* < 0.01, respectively. ns indicates not significant. Different letters above the vertical bars indicate significant differences at *p* < 0.05 according to Duncan’s multiple range test.

The main effect of light significantly impacted the RuBisCO activity and photosynthetic products. Similarly, the PBZ application significantly influenced the RuBisCO activity and soluble-sugar content. The RuBisCO activity and starch content were dramatically decreased, while the soluble-sugar content increased in the CKP treatment compared to the control. On the contrary, when sprayed with PBZ under low light, the RuBisCO activity, starch, soluble sugar, and soluble protein increased by 154.31%, 26.60%, 40.46%, and 4.51%, respectively (Figure 3).

### 2.4. Membrane Damage and Oxidative Stress

**Figure 4:**
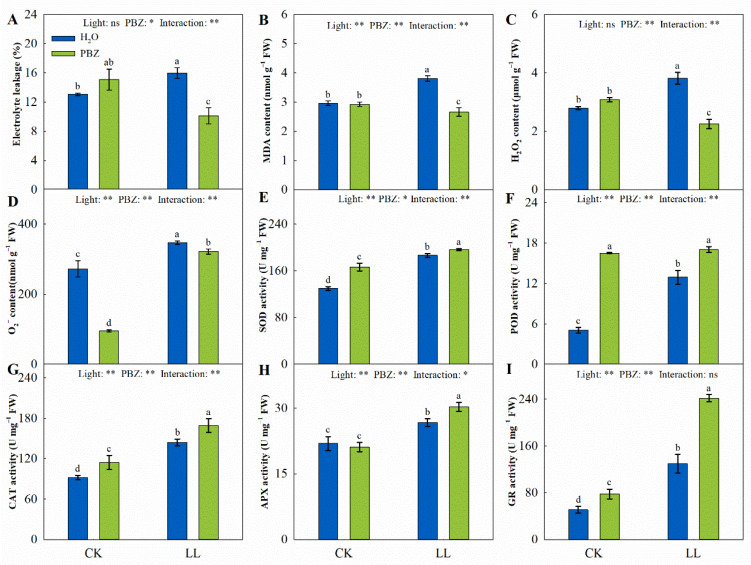
Changes in electrolyte leakage (EL, (**A**)), MDA content (**B**), H_2_O_2_ content (**C**), superoxide radical content (O_2_∙^−^, (**D**)), superoxide dismutase (SOD) activity (**E**), peroxidase (POD) activity (**F**), catalase (CAT) activity (**G**), ascorbate peroxidase (APX) activity (**H**), and glutathione reductase (GR) activity (**I**) of tall fescue leaves induced by paclobutrazol under control (CK) and LL stress. * and ** indicate significant differences at *p* < 0.05 and *p* < 0.01, respectively. ns indicates not significant. Different letters above the vertical bars indicate significant differences at *p* < 0.05 according to Duncan’s multiple range test.

The LL stress significantly increased the electrolyte leakage, MDA concentration, H_2_O_2_, and superoxide radical (O_2_∙^−^) by 22.25%, 28.61%, 36.34%, and 27.25%, while spraying PBZ after LL stress remarkably decreased them by 36.61%, 30.14%, 40.95%, and 7.34%, respectively, compared to the control (Figure 4A–D). Moreover, following a significant decline in O_2_∙^−^, the content was observed in plants treated with PBZ under the control.

### 2.5. Antioxidant System

The activities of antioxidant enzymes, including SOD, POD, CAT, APX, and GR, were significantly different among the treatments. Compared to the CK treatment, the SOD, POD, and CAT activities increased by 43.51%, 155.84%, and 56.56%, respectively, under LL stress. Their activities also increased under PBZ treatment (Figure 4E–G). The increase in APX and GR was similar to those of SOD, POD, and CAT across treatments. Additionally, the exogenous spraying of plants with PBZ enhanced the antioxidant enzyme activity under the control.

**Figure 5:**
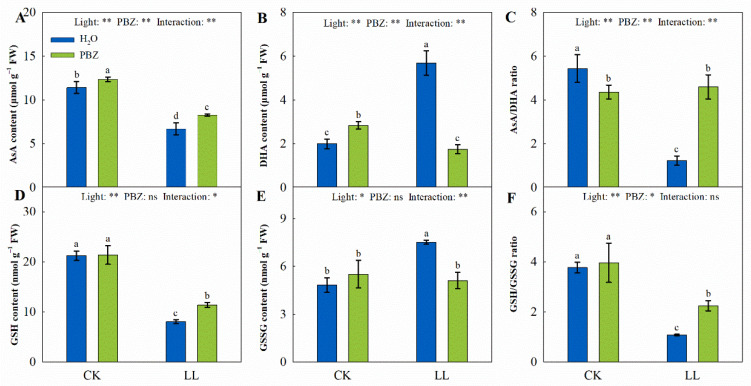
Levels of ascorbate acid (AsA) content (**A**), dehydroascorbic acid (DHA) content (**B**), AsA/DHA ratio (**C**), glutathione (GSH) content (**D**), oxidized glutathione (GSSG) content (**E**), and GSH/GSSG ratio (**F**) of tall fescue leaves induced by paclobutrazol under control (CK) and LL stress. * and ** indicate significant differences at *p* < 0.05 and *p* < 0.01, respectively. ns indicates not significant. Different letters above the vertical bars indicate significant differences at *p* < 0.05 according to Duncan’s multiple range test.

The AsA-GSH cycle is essential for activating the antioxidant system under abiotic stress. Plant exposed to LL stress recorded a significant decrease in AsA content (41.33%), while the DHA concentration was increased by 185.25% compared to the control seedlings. Eventually, the variations resulted in an evident decline of AsA/DHA under LL treatment, which caused oxidative damage in the tall fescue seedlings. In contrast, relative to the LL treatment, the ratio of AsA/DHA increased remarkably (2.73 times) following PBZ application, where the AsA concentration increased, and the DHA decreased (Figure 5). Similarly, the ratio of GSH/GSSG increased in the LLP treatment. At the same time, there was no significant difference in GSH, GSSG, and GSH/GSSG between the CKP and the control treatments. However, the AsA/DHA ratio decreased.

**Figure 6:**
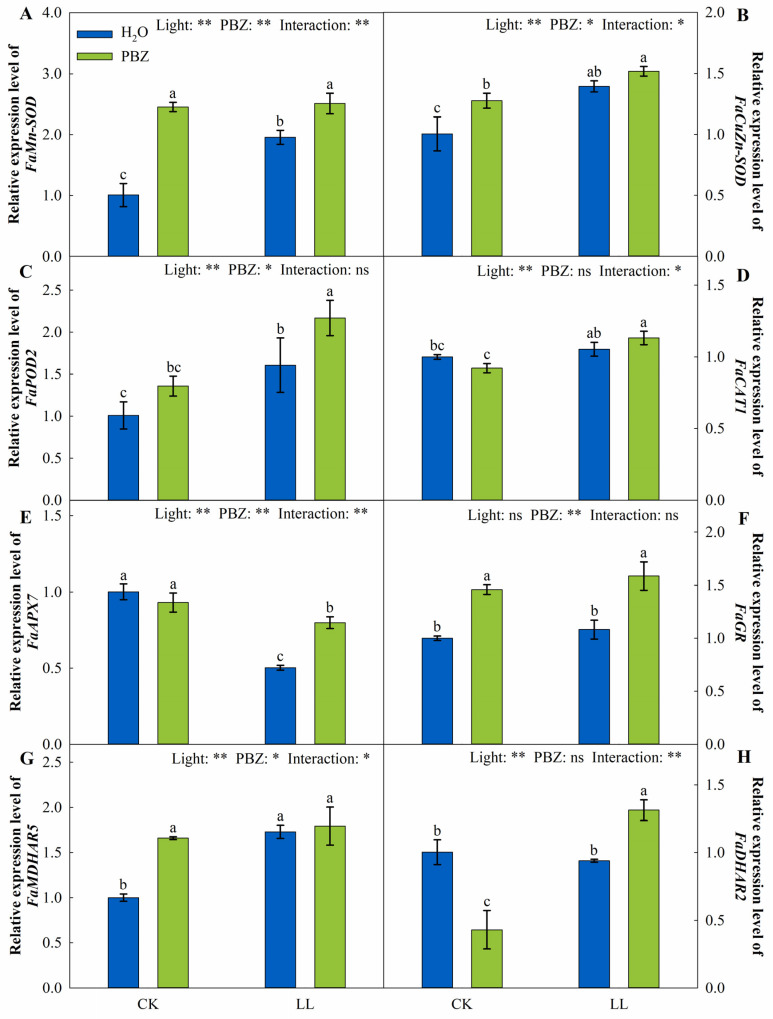
The expression level of genes encoding antioxidant enzymes of tall fescue leaves induced by paclobutrazol under control (CK) and LL stress. (**A**) *FaMn-SOD*, (**B**) *FaCuZn-SOD*, (**C**) *FaPOD2*, (**D**) *FaCAT1*, (**E**) *FaAPX7*, (**F**) *FaGR*, (**G**) *FaMDHAR5*, and (**H**) *FaDHAR2*. * and ** indicate significant differences at *p* < 0.05 and *p* < 0.01, respectively. ns indicates not significant. Different letters above the vertical bars indicate significant differences at *p* < 0.05 according to Duncan’s multiple range test.

Moreover, the transcript levels of *FaMn-SOD*, *FaCuZn-SOD*, *FaPOD2*, and *FaMDHAR5* increased by significantly 93.94%, 38.72%, 59.17%, and 72.86% under LL stress relative to the control, respectively. Nevertheless, LL treatment downregulated the expression of *FaAPX7* (Figure 6). However, the simultaneous application of PBZ and low light up-regulated *FaMn-SOD*, *FaPOD2*, *FaAPX7*, *FaGR*, and *FaDHAR2* expressions consistent with the variations in enzyme activities. Nonetheless, there was no significant change in the *FaCAT1* transcript level among the treatments (Figure 6D).

### 2.6. Endogenous GA Concentration and GA Metabolism

**Figure 7:**
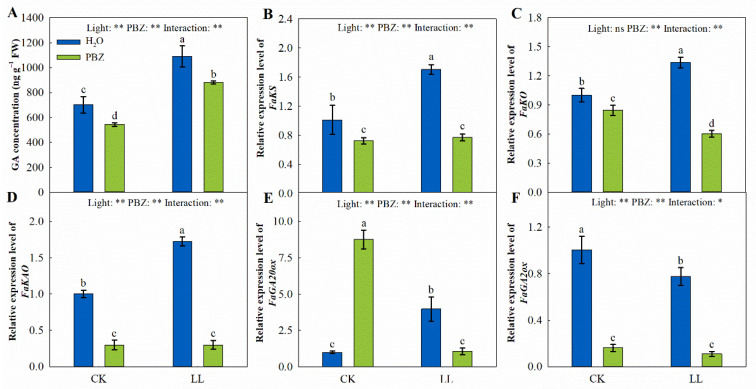
Changes in GA concentration (**A**), *FaKS* expression level (**B**), *FaKO* expression level (**C**), *FaKAO* expression level (**D**), *FaGA20ox* expression level (**E**), and *FaGA2ox* expression level (**F**) of tall fescue leaves induced by paclobutrazol under control (CK) and LL stress. * and ** indicate significant differences at *p* < 0.05 and *p* < 0.01, respectively. ns indicates not significant. Different letters above the vertical bars indicate significant differences at *p* < 0.05 according to Duncan’s multiple range test.

Endogenous GA concentrations and the genes encoding the GA biosynthesis and degradation pathways were significantly different under LL stress and the application of PBZ. Specifically, the LL treatment markedly increased the GA concentration by 55.28% and up-regulated *FaKS*, *FaKO*, *FaKAO*, and *FaGA20ox* expressions by 68.34%, 33.65%, 72.21%, and 296.22%, respectively, compared to the control (Figure 7). However, the PBZ treatment significantly decreased the GA concentration and downregulated the *FaKO*, *FaKAO*, and *FaGA20ox* transcript levels under LL stress. Moreover, plants treated with PBZ under the control recorded a lower GA concentration and transcript levels.

The expression level of *FaGA2ox*, which encodes GA degrading enzyme, was downregulated under LL stress, particularly when PBZ was added to the low-light treatment. Compared to the control, LL-induced stress downregulated the expression of *FaGA2ox* by 22.79%, which was further downregulated to 89.04% with PBZ application (Figure 7). This indicated that PBZ inhibited GA biosynthesis instead of promoting its degradation under LL stress and the control.

### 2.7. Endogenous ABA Concentration and ABA-Related Genes Expression

**Figure 8:**
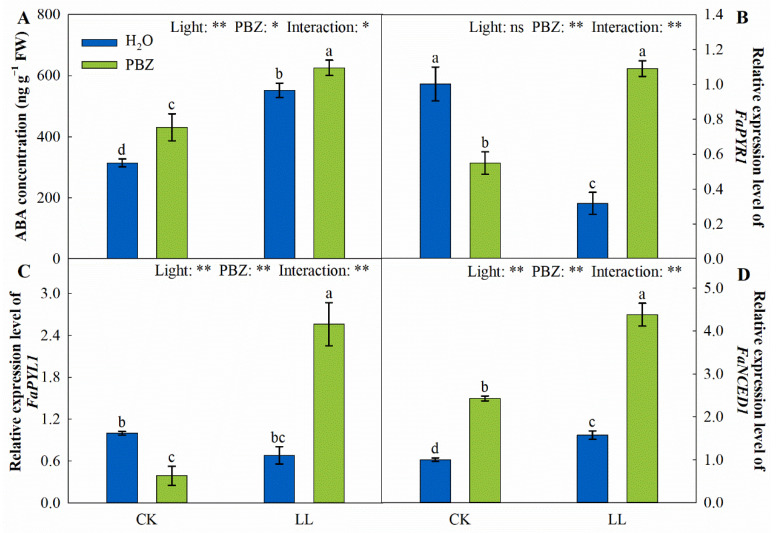
Changes in ABA concentration (**A**), *FaPYR1* expression level (**B**), *FaPYL1* expression level (**C**), and *FaNCED1* expression level (**D**) of tall fescue leaves induced by paclobutrazol under control (CK) and LL stress. * and ** indicate significant differences at *p* < 0.05 and *p* < 0.01, respectively. ns indicates not significant. Different letters above the vertical bars indicate significant differences at *p* < 0.05 according to Duncan’s multiple range test.

LL stress promoted the biosynthesis of ABA and significantly increased the endogenous ABA concentration (Figure 8). Compared to the control, the LL treatment increased the ABA concentration by 75.50% and the *FaNCED1* expression by 1.57 times, respectively. However, the highest ABA and *FaNCED1* were recorded in plants treated with PBZ under LL stress. Under the control, the PBZ application had a similar regulatory effect on ABA biosynthesis.

Quantitative real-time PCR (qRT-PCR) analyses of the relative transcript levels of *FaPYR1* and *FaPYL1* revealed dramatic down-regulation in plants exposed to LL stress, which recorded higher ABA concentrations. Compared to the control, the exogenous spraying of PBZ under the control also downregulated *FaPYR1* and *FaPYL1* by 45.18% and 61.06%, respectively. On the contrary, there was a significant increase in *FaPYR1* and *FaPYL1* by 3.42 and 3.74 times on plants simultaneously treated with PBZ and low light, respectively (Figure 8C,D).

## 3. Discussion

Light significantly affects plant growth and developmental processes. In turfgrass, these processes are impeded by a PPFD deficit and LL stress [19]. LL stress also inhibits photosynthesis, so that it cannot provide energy and carbohydrate for plant bioactivities, such as biomass accumulation and morphogenesis. Therefore, in the present study, morphological parameters, including plant height, tiller numbers, and biomass, were inhibited under LL stress (Figure 1). Similarly, in *Poa supina*, the daily growth rate and total biomass decreased under LL stress [20]. However, PBZ noticeably improved the morphological characteristics of tall fescue under LL stress (Figure 1), implying that PBZ induced LL tolerance via phenotypic alterations [21]. On the basis of morphological changes, our study elucidated more about the functions of PBZ at the physiological and molecular levels under LL stress.

Low light limits photosynthesis directly or indirectly. Directly, low irradiance influences the photosynthetic apparatus, including the alteration of the chloroplast ultrastructure, photosynthetic pigments metabolism, and photosynthetic and photochemical efficiency. Indirectly, light insufficiency negatively affects physiological characteristics, influencing photosynthesis in plants, such as photosynthetic enzymes and products [22]. PBZ stimulates cytokinin synthesis, which induces chlorophyll biosynthesis manifested as elevated chlorophyll content [12]. In the present study, applying PBZ up-regulated Chl contents compared to the control and LL treatment (Figure 2A–D). These results are consistent with previous studies on the influence of PBZ on zoysiagrass (*Zoysia japonica* Steud.) and sugar beet (*Beta vulgaris* L.) under drought stress, where PBZ up-regulated Chl and Car, which stimulated photosynthesis [23,24].

Photosynthetic pigments harvest light, affecting *P*_n_ and photochemical conversion efficiency in the photosynthetic system [25]. A decrease in *P*_n_ induced by abiotic stress is predominantly due to stomatal and non-stomatal limitations [22]. Stomatal closure retards the CO_2_ absorption by the mesophyll cells. The inhibition in CO_2_ assimilation decreases the performance of enzymes in the Calvin cycle, subsequently decreasing *P*_n_. In this study, the significant declines in *P*_n_, *g*s, and *E* were recorded under LL stress, whereas the *C_i_* increased (Figure 2E–H). Thus, LL stress mainly caused non-stomatal limitations in tall fescue. This is consistent with the previous findings of Mohan et al. [26], where PBZ application on mulberries (*Morus alba* L.) exposed to water stress alleviated the inhibition of gas-exchange parameters.

**Figure 9:**
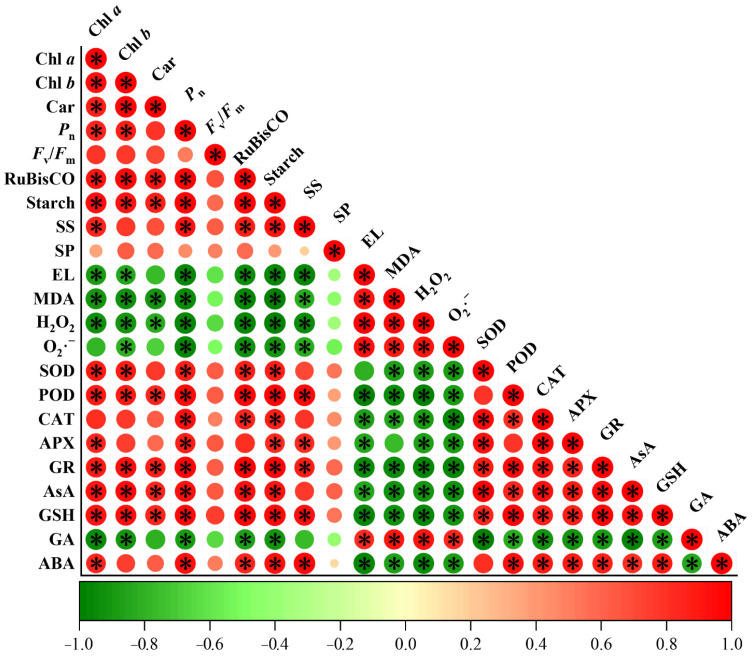
Pearson’s correlation matrix among photosynthesis, antioxidant activity, and phytohormone attributes under LL stress with and without PBZ application. Correlations are displayed by specific colors. * indicates significant differences at *p* < 0.05. Chl *a* = chlorophyll *a*, Chl *b* = chlorophyll *b*, Car = carotenoid, *P*_n_ = Net photosynthetic rate, *F*_v_/*F*_m_ = maximum quantum yield of photosystem II photochemistry, SS = soluble sugar, SP = soluble protein, EL = electrolyte leakage, MDA = malondialdehyde, H_2_O_2_ = hydrogen peroxide, O_2_·^−^ = superoxide radical, SOD = superoxide dismutase, POD = peroxidase, CAT = catalase, APX = ascorbate peroxidase, GR = glutathione reductase, AsA = ascorbic acid, GSH = reduced glutathione, GA = gibberellic acid, ABA = abscisic acid.

Furthermore, the activity of PSII influenced by Chl content, light quantum-absorption, and light-capture capacities is important during photosynthesis [27]. *F*_v_/*F*_m_ is a fluorescence parameter that indicates the conversion efficiency of light in the PSII reaction center. Thus, plants better adapt to LL environments at higher levels of *F*_v_/*F*_m_ and ΦPSII [28]. On the other hand, q_p_ is the proportion of open PSII, whose increase increases the rate of electrons transported from PSII to plastoquinone, especially to Q_A_ [29]. In this study, *F*_v_/*F*_m_, ΦPSII, q_p_, and ETR were decreased under LL treatment but up-regulated after supplementation with PBZ (Figure 2I–L). Meanwhile, the amelioration effect of PBZ on photosynthesis under LL stress was manifested by a positive correlation between chlorophyll contents, *P*_n_, and *F*_v_/*F*_m_ (Figure 9). Similar results were observed in pepper (*Capsicum annuum* L.) under simultaneous LL and PBZ treatment [30], implying that PBZ induces the activity and efficiency of the PSII reaction center to alleviate LL damage on photosynthesis.

The reduction of the photosynthetic rate under LL stress is associated with decreased concentrations and activities of enzymes used during photosynthesis. Tall fescue is a C_3_ plant that is more tolerant to LL stress than C_4_ plants. However, it is inherently not as efficient in CO_2_ assimilation as C_4_ plants [31]. RuBisCO activity limits the CO_2_ assimilation rate, especially when CO_2_ is insufficient [32]. When exposed to LL stress, biochemical adjustments are rapidly implemented to improve the light-absorption efficiency using chloroplasts. At the same time, RuBisCO activity is inhibited, since insufficient RuBP cannot saturate RuBisCO catalytic sites [33]. In the present study, the LL-related inhibition of RuBisCO activity and the mitigation of PBZ application was observed (Figure 3A). Light is necessary for the biosynthesis of photosynthetic products, which promotes biomass production in plants. Starch and soluble sugar are the carbohydrate accumulation products during photosynthesis, where lower levels of consumption of carbohydrates resulting from plant growth maintain a high photosynthetic rate [34]. Severe inhibition of carbon fixation has been observed in high-light-stressed wild-type *Arabidopsis thaliana*, which manifested as the absence of starch accumulation [35]. In addition, the decrease in starch and soluble-sugar contents in lettuce (*Lactuca sativa* L.) under LL stress also decreased the photosynthetic products [36]. In the present study, PBZ-induced increments in starch, soluble sugar, and soluble protein under LL stress were positively correlated to the RuBisCO activity (Figure 9). Thus, given the high *g*s and *F*_v_/*F*_m_ levels, PBZ might play a crucial role in maintaining photosynthesis by regulating both stomatal and non-stomatal factors under LL stress.

Membrane lipid peroxidation is the major cause of oxidative damage in plants exposed to abiotic stresses [37]. Specifically, LL stress primarily causes the generation of ROS, such as O_2_∙^−^ and H_2_O_2_, which consequently induces a lipid peroxidation and membrane permeability increase, and ultimately triggers the occurrence of oxidative stress [38,39]. In our present study, the level of ROS is remarkably increased when plants are exposed to LL stress, but exogenous PBZ application attenuates this effect. A previous study also revealed that different concentrations of PBZ decrease the EL and MDA content under sufficient-water-supply or water-deficit conditions [40]. However, in this study, the PBZ application on tall fescue under the control did not affect the extent of membrane damage and oxidative stress, but the O_2_∙^−^ level varied significantly (Figure 4). These results imply that the mitigating effect of PBZ on oxidative damage was only felt under LL stress in tall fescue.

The excessive generation of ROS-damage plant metabolism causes protein degeneration and, consequently, results in cell death [41]. Additionally, ROSs are cytotoxic, which harmfully impacts photosynthetic efficiency and electron transport without defensive systems. The physiological detoxification processes, such as antioxidant enzymes and non-enzymatic antioxidants, counteract ROS-induced damage in plants [42]. During enzymatic detoxification, SOD, a major ROS scavenger, first catalyzes O_2_∙^−^ into H_2_O_2_, and, finally, H_2_O_2_ is converted to H_2_O by POD, CAT, and APX [10]. Herein, the activities of these enzymes were remarkedly elevated under LL treatment and were further elevated after PBZ application (Figure 4). In addition, PBZ significantly enhanced the expression level of genes encoding these enzymes, which promoted ROS scavenging (Figure 6). The protective role of PBZ application on antioxidant enzymes has also been reported in wheat (*Triticum aestivum* L.) and mango (*Mangifera indica* L.) under various environmental stresses [43,44].

Moreover, the AsA-GSH cycle is responsible for H_2_O_2_ elimination in plant-cell protection [45]. AsA and GSH are pivotal molecules in the cycle, whose synergistic effect is vital for redox balance and ROS detoxification [46]. AsA is oxidized to DHA, a reaction catalyzed by APX, whereas DHA is reduced to AsA in the presence of GSH. At the same time, GSSG is converted to GSH under the catalysis of GR. In this study, when tall fescue was exposed to LL stress, AsA, GSH, AsA/DHA, and GSH/GSSG increased under PBZ treatment (Figure 5). In addition, the transcript levels of genes in the AsA-GSH cycle, including *FaGR*, *FaMDHAR5*, and *FaDHAR2*, also increased (Figure 6). This was consistent with findings by Sofy et al. [47], who reported the defensive function of PBZ under salinity stress by downregulating AsA and GSH pools. In addition, there was a strong positive correlation between AsA and GSH with antioxidant enzymes (Figure 9), which suggests that the synergic effect of the enzymatic antioxidants and the AsA-GSH cycle had a significant impact on the PBZ-induced LL tolerance.

Given PBZ alters the concentration of some important hormones, there is a likelihood that the effect of PBZ on LL stress is related to hormone metabolism [48]. Previous research indicated that GA negatively affects plants’ tolerance of various abiotic stresses, including LL stress [49]. The increase of GA can mediate shade-avoidance so that plants consume more energy fixed by photosynthesis, which decreases the photosynthetic efficiency and defense capacity [50]. Therefore, the inhibition of GA reserves the photosynthetic products, reinforcing tolerance to LL stress. In this study, the GA concentration in tall fescue was increased under LL stress, consistent with the findings on the petioles of *Arabidopsis thaliana* [51].

The GA biosynthesis in plants is performed from geranylgeranyl diphosphate and catalyzed by many enzymes, such as kaurene oxidase (KO), kaurenoic acid oxidase (KAO), and GA 20-oxidases (GA20ox), which ultimately generate bioactive GA_4_ and GA_1_ [52]. In the current study, *FaKS*, *FaKO*, *FaKAO*, and *FaGA20ox* expressions decreased under PBZ application, which weakened the GA biosynthesis, reducing the GA concentration (Figure 7). The downregulation of these genes has also been reported under high-temperature conditions following PBZ application [53]. However, the transcript level of *FaGA2ox* (a GA degrading gene) was also downregulated, implying that PBZ-mediated LL tolerance enhancement relies on the inhibition of GA biosynthesis rather than promoting its degradation.

**Figure 10:**
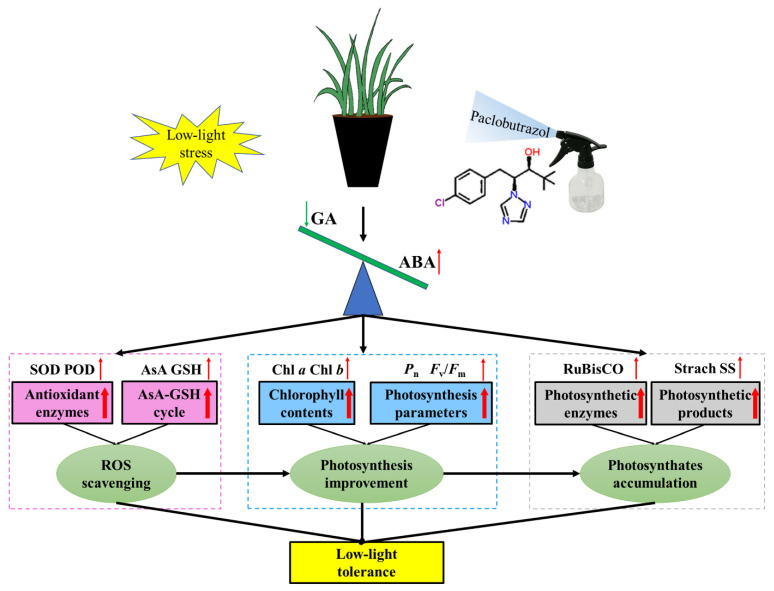
The diagram of PBZ in improving LL tolerance of tall fescue seedlings. SOD = superoxide dismutase, POD = peroxidase, AsA = ascorbic acid, GSH = reduced glutathione, Chl *a* = chlorophyll *a*, Chl *b* = chlorophyll *b*, *P*_n_ = Net photosynthetic rate, *F*_v_/*F*_m_ = maximum quantum yield of photosystem II photochemistry, SS = soluble sugar. Red and green arrows indicated rise and fall, respectively.

Abscisic acid (ABA), a plant hormone regulating plant growth and development, also acts as a signaling molecule in plant responses to stress [54]. The stress-resistance mediated by PBZ might be due to changes in hormones, including cytokinin and ABA [55]. Thus, it was known that the stress-protection effect of PBZ might be derived from ABA. The biosynthesis of ABA is regulated by 9-*cis*-epoxycarotenoid dioxygenase (NCED), which is a rate-limiting step [56]. In the current study, PBZ up-regulated the ABA-receptor genes (*FaPYR1* and *FaPYL1*) and ABA synthesis gene (*FaNCED1*), thereby increasing the endogenous ABA concentration under LL stress (Figure 8). This is consistent with previous findings by Opio et al. [57], who identified the upregulation of the ABA biosynthesis-related gene *NECD1* and the transporter gene *AITb-like* under PBZ treatment. Moreover, the application of ABA directly protects tall fescue against LL stress by activating ROS scavenging and enhancing the photosynthetic performance [38]. Therefore, PBZ enhanced the LL tolerance of tall fescue seedlings by enhancing the ABA concentration to activate the downstream response (Figure 10).

Overall, the PBZ treatment induced ABA metabolism while inhibiting GA metabolism. On the one hand, an increased ABA concentration enhances the photosynthetic performance and antioxidant capacity to remove excess ROS, as indicated by increased Chl *a* content, Chl *b* content, *P*_n_, *F*_v_/*F*_m_, RuBisCO activity, antioxidant enzyme activities, and antioxidant contents. On the other hand, a decreased GA concentration reduces carbon consumption and promotes the accumulation of photosynthetic products, including starch and soluble-sugar and soluble-protein content. In conclusion, the findings in this study reveal that PBZ mediates the actions of GA and ABA, consequentially strengthening LL stress tolerance in tall fescue seedlings.

## 4. Materials and Methods

### 4.1. Plant Materials and Growth Conditions

Tall fescue (*Festuca arundinacea* Schreb. cv. Arid3) seeds were obtained from Beijing Clover Seed & Turf Co., Beijing, China. The seeds were sterilized with 0.1% (*w*/*v*) sodium hypochlorite for 10 min and then washed with sterile water thrice, followed by germinating on the surface of moistened filter paper for 7 d. Next, eight seedlings with uniform growth were transplanted into a lightproof plastic pot (9 cm in diameter, 15 cm in height), filled with sterilized quartz sand. Finally, all seedlings were incubated in a climate chamber at 25/20 °C (day/night), relative humidity of 60/50% (day/night), and a photoperiod of 16/8 h (light/night cycle) with a photosynthetic photon flux density (PPFD) of 500 μmol m^−2^ s^−1^ above the plants. The plants were watered every day and irrigated with Hoagland’s nutrient solution (pH 6.5, adjusted by citric acid) every three days. The seedlings were precultured for 35 d.

### 4.2. Experimental Designs and Treatments

Experiment 1: To determine the most effective PBZ concentration in alleviating LL stress, the 35-day-old tall fescue seedlings were randomly divided into six groups; each group contained three pots; and each group was randomly treated with different concentrations of PBZ under LL stress for 14 d, including 0, 50, 100, 200, 300, and 500 mg L^−1^. In this case, each treatment was replicated thrice. During the 14 d treatment, the PPFD above the plants was 40 μmol m^−2^ s^−1^, according to our previous research on different low-light intensity ranges [58,59], and the PBZ was sprayed four times. According to the comprehensive results of the morphology, the total chlorophyll content, and the MDA content, the optimal PBZ concentration was identified (Table 1).

Experiment 2: To further evaluate the effect of PBZ in regulating LL stress, all the 35 d old seedlings were randomly assigned into four groups; each group contained three pots. The treatments were administered as follows: (1) Control (CK): distilled water under normal light (500 μmol m^−2^ s^−1^ PPFD); (2) CKP: 200 mg L^−1^ PBZ under normal light; (3) LL: distilled water under LL stress (40 μmol m^−2^ s^−1^ PPFD); and (4) LLP: 200 mg L^−1^ PBZ under LL stress. Each group was randomly treated with one of the above treatments. PBZ was sprayed four times during a 14 d treatment period. After the 14 d treatment, the third fully expanded leaf samples from the top were randomly collected and immediately frozen in liquid N_2_ and stored at −80 °C for subsequent biochemical analysis.

### 4.3. Measurements of Growth and Morphological Parameters

At the end of the 14 d treatment, plant height and leaf width were determined using a vernier caliper. The number of tillers per plant was also counted. Next, the whole plants were uprooted and washed with double distilled water to remove the adhering quartz-sand particles. Finally, the plants were oven-dried at 80 °C for 72 h, and their total dry biomasses were determined.

### 4.4. Determination of Chlorophyll Content, Photosynthesis, and Chlorophyll Fluorescence Parameters

Fresh tall fescue leaves (0.1 g) were randomly harvested and soaked in 5 mL of 95% ethyl alcohol and 10 mL of 80% acetone for 24 h in the dark [60]. The absorbance of the extract was measured using a spectrophotometer (UV-1800, MAPADA, Shanghai, China) at 440, 645, and 663 nm to determine the chlorophyll *a* (Chl *a*), chlorophyll *b* (Chl *b*), total Chl, and carotenoid (Car) concentrations following the method of Arnon [61].

Gas-exchange parameters, including the net photosynthetic rate (*P*_n_), stomatal conductance (*g*s), intercellular CO_2_ concentration (*C_i_*), and transpiration rate (*E*), were also measured on the third fully expanded leaf from the top using a Li-6400XT portable photosynthesis system (LI-COR, Lincoln, NE, USA) from 9:00 to 11:00 a.m. In addition, the chlorophyll fluorescence parameters were measured after 30 min of dark-adaptation (using a leaf clip) using a Mini-PAM fluorometer (PAM2500, Walz, Effeltrich, Germany) on the same leaf where *P*_n_ was measured. The maximum quantum yield of photosystem II (PSII) photochemistry (*F*_v_/*F*_m_), the actual quantum yield of PSII photochemistry (ΦPSII), the photochemical quenching coefficient (q_p_), and the relative electron transport rate (ETR) were calculated according to the formulas previously formulated [62].

### 4.5. Quantification of RuBisCO Activity and Photosynthetic Products

The leaf RuBisCO activity was estimated using an assay kit purchased from Solarbio Life Sciences (BC0440, Beijing, China), following the manufacturer’s instructions. One unit of RuBisCO activity was defined as 1 nmol NADH oxidized per one gram of leaf at 25 °C for one min. The extraction and calculation of the starch content in the leaves were performed following the method described by Barrios et al. [63]. The soluble protein concentration was determined using the Bradford method [64]. The soluble sugar was quantified using the anthrone colorimetric method as previously described [65].

### 4.6. Estimation of the Ion Leakage and Oxidative Damage

The electrolyte leakage (EL) was quantified as previously described, with some modifications [66]. Fresh leaves (0.5 g) were washed and incubated in 10 mL of deionized water for 1.5 h at 25 °C, after which conductivity (S_1_) was measured. Next, the samples were heated in boiling water for 10 min, cooled at room temperature, and conductivity was measured again (S_2_). The EL was calculated as S_1_/S_2_ × 100%.

In addition, 0.5 g leaves were ground in 5 mL 10% (*w*/*v*) trichloroacetic acid (TCA) to extract malondialdehyde (MDA). The MDA concentration was determined by mixing 2 mL sample extract with 2 mL thiobarbituric acid, then incubating in boiling water for 15 min. The mixture was then centrifuged at 3000 r/min for 10 min, and the absorbance of the supernatant was measured at 450, 532, and 600 nm using a spectrophotometer [67].

Hydrogen peroxide (H_2_O_2_) was also measured using the titanium sulfate (TiSO_4_) method with slight modification [68]. The frozen leaves were homogenized with precooling acetone and centrifuged at 5000× *g* for 20 min at 4 °C. Next, the sediment was washed three times with acetone, and 5% (*w*/*v*) TiSO_4_ and 2 M H_2_SO_4_ were added, respectively. Finally, the absorbance was measured at 415 nm using a spectrophotometer.

To determine the concentration of the superoxide radical (O_2_∙^−^), O_2_∙^−^ was extracted in 50 mM phosphate buffer (pH 7.8) as previously described, with some modifications [69]. The reaction system consisted of 50 mM phosphate buffer, 10 mM hydroxylamine hydrochloride, 17 mM sulphanilic acid, 7 mM α-naphthyl amine, and sample extract. Finally, the O_2_∙^−^ concentration was determined by measuring the absorbance at 530 nm using a spectrophotometer.

### 4.7. Determination of the Activity of the Antioxidant Enzymes

The frozen samples were ground in 50 mM phosphate buffer (pH 7.8), containing 1 mM ethylenediaminetetraacetic acid and 1% polyvinyl pyrrolidone, until homogenized. The homogenate was then centrifuged at 15,000× *g* for 20 min at 4 °C for enzyme extraction. The superoxide dismutase (SOD) activity was determined in a reaction mixture consisting of 14.5 mM methionine, 3 mM sodium ethylene diamine tetraacetate (EDTA-Na_2_), 60 μM riboflavin, 2.25 mM nitroblue tetrazolium (NBT), and the enzyme extract [70]. One unit of SOD activity equated to the 50% inhibition of NBT photoreduction. Peroxidase (POD) activity was measured based on the oxidation of guaiacol by H_2_O_2_ [71]. The catalase (CAT) activity was measured using an assay kit (BC0200, Solarbio, Beijing, China) according to the manufacturer’s instructions. The ascorbate peroxidase (APX) activity was measured in terms of the method of Nakano and Asada [72]. Each unit of APX was defined as 1 μmol ascorbic acid (AsA) oxidized in the presence of H_2_O_2_ for 1 min. The glutathione reductase (GR) activity was measured in a reaction system consisting of 10 mM NADPH, 10 mM oxidized glutathione (GSSG), and the enzyme extract. One unit of GR was defined as 1 μmol NADPH oxidized per 1 g for 1 min [73].

### 4.8. Determination of Non-Enzymatic Antioxidants

The AsA and dehydroascorbic acid (DHA) concentrations were determined according to the bathophenanthroline (BP) method previously described [74]. Tall fescue leaf samples (1.0 g) were ground in liquid N_2_ and immediately transferred into 5 mL of 5% (*w*/*v*) metaphosphoric acid. The homogenate was centrifuged at 12,000× *g* for 15 min at 4°C to extract AsA. A portion of the supernatant was collected and mixed with deionized water. To the remaining supernatant, 10 mM dithiothreitol and 0.5% (*w*/*v*) N-ethylmaleimide were added in equal volumes. Next, 10% (*w*/*v*) TCA, 44% (*v*/*v*) phosphoric acid, 0.5% (*w*/*v*) BP-ethyl alcohol, and 0.3% (*w*/*v*) ferric trichloride were added to these two supernatants. The concentrations of AsA and total ascorbic acid were obtained by measuring the absorbance of the two reaction mixtures at 525 nm, respectively. The DHA content was calculated by subtracting the AsA from the total ascorbic acid concentration.

Moreover, the glutathione (GSH) and GSSG concentrations were detected and calculated using the 5,5′-dithiobis-(2-nitrobenzoic acid) (DTNB) method [75]. Tall fescue leaf samples (2.5 g) were ground to powder, followed by extraction in 5 mL of 50 g L^−1^ TCA (containing 5 mM EDTA-Na_2_). The extraction solution was centrifuged at 12,000× *g* for 20 min at 4 °C to collect the supernatant. Next, the extract and 4 mM DTNB were mixed up, and their absorbance at 540 nm was recorded to estimate GSH. Then, GR was added to reduce GSSG to GSH; thus, the GSSG concentration was calculated by subtracting the GSH from the total glutathione concentration.

### 4.9. Estimation of GA and ABA Concentrations

The GA and ABA in the leaf tissues were extracted and detected using a liquid chromatography-mass spectrometry (LC-MS) system as previously described, with some modifications [76]. The leaf samples (0.1 g) were crushed in liquid nitrogen using a mortar and pestle. The ground powder was soaked in acetonitrile and 1% acetic acid overnight at 4 °C for extraction, followed by vortexing for 2 h and centrifugation at 10,000× *g* for 10 min at room temperature. The supernatant was lyophilized, dissolved in 100 μL of 70% (*v*/*v*) aqueous methanol, and centrifuged at 16,000× *g* for 5 min at room temperature before the LC/MS analysis. Finally, the GA and ABA concentrations were analyzed through a rapid-resolution liquid chromatography system (Agilent 1200, Santa Clara, CA, USA)—tandem electrospray ionization triple quadrupole mass spectrometer system (Agilent 6460). Multiple-reaction monitoring was used for MS analysis, and the analytic parameters were set as described by Li et al. [77].

### 4.10. Quantitative Real-Time PCR (qRT-PCR) Analysis

The total RNA was extracted using an E.Z.N.A.^®^ Plant RNA Kit (Omega Bio-tek, Norcross, GA, USA), according to the manufacturer’s protocol. High-quality RNA (1 μg) was reverse transcribed into cDNA in a 20 μL reaction using a HiScript^®^II 1st Strand cDNA Synthesis Kit (Vazyme, R211, Vazyme Biotech, Nanjing, China), according to the manufacturer’s protocol. To detect the transcript changes of genes under different treatments, qRT-PCR was conducted using ChamQ Universal SYBR qPCR Master Mix (Vazyme, Q711), following the manufacturer’s protocol. Gene-specific primers were designed according to transcript sequence and are listed in Table S1. The relative expression levels of the genes were calculated by the 2^−ΔΔCt^ method [78] using *FaActin* as the internal control.

### 4.11. Statistical Analysis

Each experiment was arranged in a completely randomized design with three biological replicates. The statistical data were subjected to a two-way ANOVA analysis in the SPSS 22 (SPSS Inc., Chicago, IL, USA). All data were expressed as means ± SD. The differences between the means were separated using Duncan’s multiple range test with *p* < 0.05, indicating significant differences between the means. Pearson’s correlation analyses determined the correlations between the photosynthetic and physiological indexes with the foliar PBZ application under LL stress.

## Figures and Tables

**Table 1:** Effects of different paclobutrazol (PBZ) concentrations (0, 50, 100, 200, 300, 500 mg L^−1^) on physiological parameters of tall fescue. Data presented are mean ± SD (n = 3). Different letters next to the number indicate a significant difference at *p <* 0.05 (Duncan’s multiple range test). CK, control treatment with distilled water; LL, treatment with distilled water under low-light stress; LL + 50, treatment with 50 mg L^−1^ PBZ under LL stress; LL + 100, treatment with 100 mg L^−1^ PBZ under LL stress; LL + 200, treatment with 200 mg L^−1^ PBZ under LL stress; LL + 300, treatment with 300 mg L^−1^ PBZ under LL stress; LL + 500, treatment with 500 mg L^−1^ PBZ under LL stress.

Treatments	Plant Height (cm)	Leaf Width (mm)	Total Biomass (g plant^−1^)	Total Chl Content (mg g^−^^1^ FW)	MDA Content (nmol g^−1^ FW)
CK	26.27 ± 3.92 a	4.75 ± 0.05 a	0.59 ± 0.06 a	3.73 ± 0.24 a	2.46 ± 0.05 c d
LL	16.53 ± 1.36 c	2.74 ± 0.09 c	0.09 ± 0.02 c	2.32 ± 0.04 d	2.73 ± 0.04 b
LL + 50	16.90 ± 1.31 c	3.36 ± 0.19 c	0.09 ± 0.01 c	2.24 ± 0.17 d	2.63 ± 0.14 b c
LL + 100	15.64 ± 3.06 c	3.50 ± 0.16 c	0.08 ± 0.03 c	2.59 ± 0.09 c	2.93 ± 0.04 a
LL + 200	21.07 ± 0.51 b	3.78 ± 0.16 b	0.16 ± 0.01 b	3.01 ± 0.15 b	2.30 ± 0.15 d
LL + 300	17.14 ± 2.45 c	3.24 ± 0.08 c	0.08 ± 0.02 c	2.79 ± 0.05 c	2.66 ± 0.14 b
LL + 500	16.14 ± 1.88 c	2.99 ± 0.16 c	0.09 ± 0.01 c	2.28 ± 0.10 c	2.62 ± 0.02 b c

## Data Availability

The data presented in this study are available on request from the corresponding author.

## Supplementary Materials

The supporting information can be downloaded at: https://www.mdpi.com/article/10.3390/ijms23179966/s1.
