# Supplementary material for: Paclobutrazol Ameliorates Low-Light-Induced Damage by Improving Photosynthesis, Antioxidant Defense System, and Regulating Hormone Levels in Tall Fescue
Source: Int J Mol Sci. 2022 Sep 1;23(17):9966. doi: 10.3390/ijms23179966 (PMC9456200; doi:10.3390/ijms23179966)
Supplement: Supplementary file 1 [file ijms-23-09966-s001.zip › ijms-1882657-supplementary.pdf]

Table S1

Sequences of primers used in quantitative real-time PCR (qRT-PCR) test.

Gene name	Forward primer (5' to 3')	Reverse primer (5' to 3')
FaActin	TCTTACCGAGAGAGGTTACTCC	CCAGCTCCTGTTCATAGTCAAG
FaKS	ACGGAAGGCTAGAATAAGGAAGCA	TCGTAGTCCTCTCCTGATATGCTCC
FaKO	TGTTCCCGGTTTACCGCTTATT	GCTGAGTTGAGCACAGCTACAGATG
FaKAO	CTTCCTCCGCGCCTTCAAGT	TGTCACTACCATGATCGTCGGG
FaGA20ox	TCAACATCGGCGACACATTCAT	CAGAGGAAGTAAGCCAGCGACC
FaGA2ox	GATGTGACGCAGGAGGTAGCGA	TTCAGCCGGAGGAAGCACGT
FaPYR1	TACAAGCACTTCATCCGCAGCT	GGTGATGGTGAAGCCGAAGG
FaPYL1	ACTTCATCAAGTCCTGCGACCTC	GACGACGCTGAAGCTGAGGAT
FaNCED1	CTCTTCGACGGTGACGGCAT	TGAAAGCCGGAGAAGACGTTG
FaMn-SOD	CACCTACGTTGCCAACTACAACAAG	CTCATTAGCAGGCTTGAGGTTCTTC
FaCuZn-SOD	TCAACTGGGTACACCGAGGTGA	TCGTTCAGTATCAATAGGTGCTCCA
FaPOD2	CATGGGAAGAAAGTGGTCATCTTC	CTGCCCATCCGTTGAGAACATA
FaCAT1	CGATCCTGCTTGAGGACTACCAC	GTCGTGGGTCACCTCGAAGAAG
FaAPX7	ATGGCACGATTCTGGCACATAT	GCCAACTGGAATAAATCTGCGTA
FaGR	GGTAGGCCATCAATGCCAAATAT	GCCGAATAAACACATGAACCTCA
FaMDHAR5	CGGCTGGAATCTTGACACAACT	TCAGAGCCAGCATCAAGTTTGTC
FaDHAR2	CAGGCACTCGATGAGCACCTA	GTGTAGGCATAGACGCTGGTCAG
